# New Conjugated Benzothiazole-*N-*oxides: Synthesis and Biological Activity

**DOI:** 10.3390/molecules14125382

**Published:** 2009-12-23

**Authors:** Peter Gajdoš, Peter Magdolen, Pavol Zahradník, Pavlína Foltínová

**Affiliations:** 1 Department of Organic Chemistry, Faculty of Natural Sciences, Comenius University, Mlynská dolina CH-2, 842 15 Bratislava, Slovakia; 2 Institute of Subcellular Biology, Faculty of Natural Sciences, Comenius University, Mlynská dolina CH-2, 842 15 Bratislava, Slovakia

**Keywords:** benzothiazole, *N*-oxides, push-pull systems, antibacterial, *Euglena gracilis*

## Abstract

Eleven new 2-styrylbenzothiazole-*N*-oxides have been prepared by aldol – type condensation reactions between 2-methylbenzothiazole–*N*-oxide and *para-*substituted benzaldehydes. Compounds with cyclic amino substituents showed typical push-pull molecule properties. Four compounds were tested against various bacterial strains as well as the protozoan *Euglena gracilis* as model microorganisms. Unlike previously prepared analogous benzothiazolium salts, only weak activity was recorded.

## Introduction

**Figure 1:**
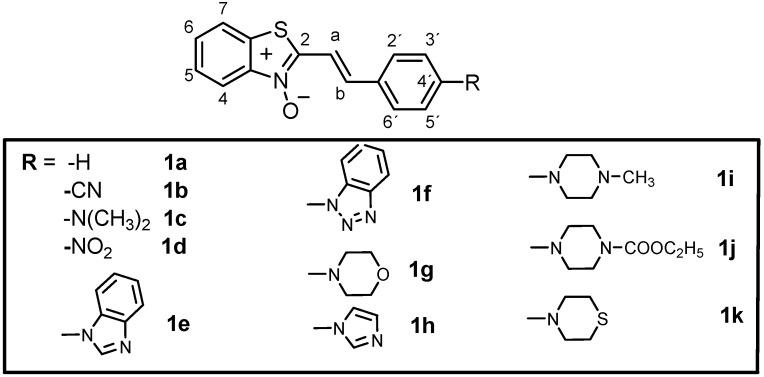
Structure of target benzothiazole-*N*-oxides.

Various benzothiazoles are known as industrial chemicals, dyes and drugs [1,2,3]. The biological activity of compounds with benzothiazole skeletons includes anticancer, antibacterial, antifungal and anthelmintic properties [4,5]. Some of the active antimicrobial substances have a dipolar push-pull structures with benzothiazole as the acceptor part of the molecule and an amino-substituted benzene ring as a donor part, linked together with a conjugated bridge. A previous QSAR study of such a type of compounds revealed that 2-styrylbenzothiazoles with *p*-dialkylamino-substituted benzene rings presented interesting antimicrobial activity [6]. The next generation of derivatives showed enhanced activity when a conjugated bridge was enlarged to two or three double bonds, the dialkylamino substituent was changed to *N*-cyclic amino and the nitrogen in benzothiazole part of molecule was quaternized to the corresponding *N-*alkylbenzothiazolium salt, preferentially with allyl, propargyl or methyl halogenides [7]. The substitution at the benzene ring of benzothiazole did not add any substantial bonus. As the quaternization of the benzothiazole nitrogen by an alkyl group improved the biological activity 2-3 fold [8,9], we shifted our interest to the benzothiazolium-*N*-oxide derivatives assuming a similar activity effect might result. The *N*-oxide structure could also influence the electron density in the benzothiazole ring what could extend the synthetic route to derivatives substituted on the heterocycle phenyl ring. Since the benzothiazole push-pull dipolar structures also show important nonlinear optical properties, photonics and optoelectronics could be other fields of possible application [9,10,11]. These were the main reasons to synthesize the target compounds with the general structure presented in Figure 1.

In this paper the synthesis and characterization of eleven new 2-styrylbenzothiazole-*N*-oxides is reported. Diverse substituents R are bonded to the 4´-position of the styryl part of the molecule: electron-acceptors –NO_2_ and –CN as well as electron-donors represented by the dimethylamino group and various *N-*cyclic amines. Four chosen compounds were also tested against various model microorganisms. 

## Results and Discussion

**Scheme 1:**
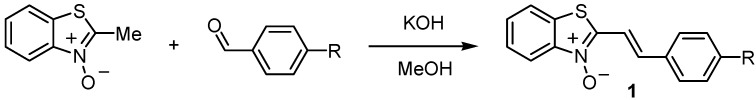
Synthesis of benzothiazole-*N*-oxides.

As the quaternization of benzothiazole heterocyclic nitrogen causes increased acidity of the 2-methyl hydrogens, the most appropriate way to prepare the target styryl compounds consists of the aldol-type condensation of 2-methylbenzothiazole-*N*-oxide and a substituted benzaldehyde (Scheme 1). Methanol was chosen as a suitable solvent due to its good solvatation of the reactants. The use of dimethylsulfoxide, which was beneficial in the case of the reaction between simple 2-methylbenzothiazole and the respective aldehydes did not show any advantage in this case as the products remain dissolved in the reaction mixture. 2-Methylbenzothiazole-*N*-oxide was prepared from 2-methylbenzothiazole by the known oxidation procedure with hydrogen peroxide–maleic anhydride mixture [12]. The required *p*-substituted benzaldehydes were synthesized via nucleophilic aromatic substitution from 4-fluorobenzaldehyde [13]. 

The prepared derivatives were identified by ^1^H- and ^13^C-NMR, UV-vis spectroscopy and elemental analysis. The physicochemical properties of prepared compounds and their biological activity were compared with the same data of analogous condensation products of 2-methyl-3-alkylbenzothiazolium salts with the corresponding aldehydes.

UV–VIS absorption spectra of the prepared compounds were measured and they consist of the long-wavelength band located between 340–420 nm and other band at shorter wavelength sometimes separable to two bands. The long wavelength band is usually the most intensive one and represents the S_0_→S_1_ electronic transition. The position of λ_max_ of this band strongly depends on the character of a substituent in the *para* position on the styrene moiety. The electron-donating cyclic amine substituents cause a higher push-pull effect, expressed with the batochromic shift of λ_max_ to 400–420 nm. These compounds appear as orange – red solids, contrary to yellow colour of the compounds with electron-accepting or heteroaromatic substituents at the same position. 

In all, of the eleven new benzothiazole derivatives prepared, four of them with the divers types of aminosubstituents were tested for their antimicrobial and antifungal activity. The activities of prepared compounds have been measured and compared with the corresponding neutral conjugated benzothiazoles and *N*-alkyl benzothiazolium salts. Unfortunately the new derivatives are less active than the analogous benzothiazolium salts. They are comparable to the corresponding neutral conjugated benzothiazole derivatives. The results are listed in Table 1. The activities against the unicellular flagelate *Euglena gracilis* were also low and did not exceed a value of 3 expressed as log(1000/ED_50_). The activities of the corresponding *N*-alkyl benzothiazolium salts were found to be more than 6 [8]. 

**Table 1:** The quantitative antibacterial and antifungal activities of new benzothiazole-*N*-oxides.

Compound	MIC values (μg/mL) for individual organisms					
	*S. aureus*	*B. subtilis*	*E. coli*	*P. aeruginosa*	*C. albicans*	*M. gypseum*
**1c**	50	500	>500	>500	250	500/250
**1e**	250	500	>500	>500	250	> 500/250
**1f**	250	500	>500	>500	250	> 500/500
**1h**	250	500	>500	>500	250	> 500/500

## Conclusions

In summary, an aldol type condensation reaction between 2-methylbenzothiazole–N-oxide and divers types of *p-*substituted benzaldehydes has been used to prepare eleven new 2-styrylbenzothiazole-*N*-oxides. The UV-vis spectra show typical push-pull character in derivatives with amino substituents. Four compounds were tested against various bacterial strains as well as the protozoan *Euglena gracilis.* However only weak activity was recorded compared with previously prepared analogous benzothiazolium salts. 

## Experimental

### General

NMR spectra were recorded on a Varian Gemini 2000 spectrometer, at 300 MHz (^1^H) and 75 MHz (^13^C) using tetramethylsilane as an internal standard. UV-vis spectra were measured on Hewlett-Packard Diode Array 8245 in methanol (UV grade) and ε values are expressed in mol^-1^·m^2^. Elemental analysis was determined on a Carlo Erba Science 1106 instruments and obtained values were in acceptable error range (0.4%). Solvents were dried and distilled before use. Condensation products were purified by crystallization. 

### 2-Methylbenzothiazole-N-oxide

To a solution of 2-methylbenzothiazole (7.5 g, 0.05 mol) and maleic anhydride (6.10 g, 0.062 mol) in CH_2_Cl_2_ (25 mL) 30% aqueous H_2_O_2_ (6.35 g, 0.18 mol) was added dropwise. The mixture was stirred and heated to reflux for 1 h, then cooled in the ice-water bath. The resulting precipitated maleic acid was filtered off and washed with CH_2_Cl_2_. The combined filtrates were dried and concentrated under reduced pressure. Chromatography of the residue on silica gel using MeOH-CH_2_Cl_2_ (1:15) gave 2-methylbenzothiazole-*N*-oxide (2.9 g, 36%) as a white solid, m.p. 46–48 °C. ^1^H-NMR (CDCl_3_) δ 8.12–8.15 ( 1H, m, 4-H), 7.96–8.00 (1H, m, 7-H), 7.58–7.70 (2H, m, 5,6-H), 2.65 (3H, s, CH_3_). 

### General procedure for the preparation of condensation products

2-Methylbenzothiazole-*N*-oxide (0.16 g, 0.001 mol) and the appropriate substituted benzaldehyde (0.001 mol) were dissolved in methanol (2 mL). Potassium hydroxide (0.05 mL of 50% aqueous solution) was added as a base catalyst and the mixture was heated to reflux for 3 h. The precipitated crude product was crystallized from ethanol to give pure compound.

*2-*[*(E)-2-Phenylvinyl*]*-1,3-benzothiazole-3-oxide* (**1a**): Yield 74%; M.p 241-244 °C; ^1^H-NMR (DMSO-d_6_) δ 8.15–8.18 (1H, m, H-7), 7.99–8.02 (1H, m, H-4), 7.89 (1H, d, *J =* 16.5, H-β), 7.76 (2H, d, *J =* 8.1, H-2,6), 7.73 (1H, d, *J =* 16.5, H-α), 7.64–7.67 (2H, m, H-5,6), 7.37–7.48 (3H, m, H-3,4,5); ^13^C- NMR: (DMSO-d_6_) δ 142.86, 141.38, 135.91, 135.51, 129.57, 128.99, 128.52, 127.55, 127.38, 125.86, 123.86, 116.81, 114.48; UV-vis: λ_max_ /nm (log *ε*): 344 (3.19). 

*2-[(E)-2-[4-Cyanophenyl]vinyl]-1,3-benzothiazole-3-oxide* (**1b**): Yield 66%. M.p. 224- 228 °C. ^1^H NMR (DMSO) δ 8.18–8.21 (1H, m, H-7), 8.01–8.04 (1H, m, H-4), 7.96 (2H, d, *J =* 8.4, H-3`,5`), 7.86–7.91 (2H, m, H-α,β), 7.89 (2H, d, *J =* 8.4, H-2`,6`), 7.66–7.69 (2H, m, H-5,6). ^13^C NMR: (DMSO) δ 142.74, 141.88, 140.09, 135.96, 133.41, 132.59, 128.72, 127.99, 127.36, 126.13, 118.55, 117.66, 116.81, 110.98. UV-vis: λ_max_ /nm (log *ε*): 347 (3.95). 

*2-[(E)-2-[4-(Dimethylamino)phenyl]vinyl]-1,3-benzothiazole-3-oxide* **(1c)**: Yield 31%. M.p. 255–259 °C. ^1^H NMR (CDCl_3_) δ 8.11–8.14 (1H, m, H-7), 7.66–7.72 (1H, m, H-4), 7.62 (1H, d, *J =* 16.4, H-β), 7.52 (2H, d, *J =* 8.9, H-2`,6`), 7.42–7.55 (2H, m, H-5,6), 7.38 (1H, d, *J =* 16.4, H-α), 6.70 (2H, d, *J =* 8.9, H-3`,5`), 3.04 (6H, s, N(CH_3_)_2_). ^13^C NMR (CDCl_3_) δ 150.95, 145.69, 141.73, 137.32, 132.98, 128.92, 127.74, 127.19, 126.55, 124.37, 116.89, 111.54, 109.25, 39.74.

*2-[(E)-2-[4-Nitrophenyl]vinyl]-1,3-benzothiazole-3-oxide* **(1d)**: Yield 54%; M.p. 230–234 °C; ^1^H- NMR: (DMSO-d_6_) δ 8.10–8.14 (1H, m, H-7), 7.94–7.97 (1H, m, H-4), 7.71 (1H, d, *J =* 16.5, H-β), 7.61–7.63 (4H, m, H-5,6, H-3`,5`), 7.51 (1H, d, *J =* 16.4, H-α), 6.99 (2H, d, *J =* 8.8, H-2`,6`); ^13^C-NMR: (DMSO-d_6_) δ 151.79, 146.68, 143.44, 141.65, 139.68, 132.17, 127.70, 126.94 (2C), 126.73, 126.63, 123.27 (2C), 116.57, 109.31; UV-vis: λ_max_ /nm (log *ε*): 276 (2.85). 

*2-[(E)-2-[4-(1H-Benzo[d]imidazole-1-yl)phenyl]vinyl]-1,3-benzothiazole-3-oxide* (**1e**): Yield 40%; M.p 186–188 °C; ^1^H-NMR: (DMSO-d_6_) δ 8.64 (1H, s, H-2_benzim_), 8.17–8.20 (1H, m, H-7), 8.00–8.05 (1H, m, H-4), 8.03 (2H, d, *J =* 8.8, H-2`,6`), 7.83 (1H, d, J = 16.7, H-β), 7.79 (2H, d, *J =* 8.8, H-3`,5`), 7.78 (1H, d, *J =* 16.7 H-α), 7.73–7.80 (2H, m, H-4,7_benzim_), 7.66–7.73 (2H, m, H-5,6), 7.29–7.42 (2H, m, H-5,6_benzim_); ^13^C-NMR (DMSO-d_6_) δ 145.48, 143.53, 143.23, 142.98, 142.82, 142.04, 133.75,132.48, 132.88, 129.13 (2C), 128.58, 127.42, 125.91, 123.73, 123.59, 122.61, 119.95, 116.78, 115.17, 110.82;UV-vis: λ_max_ /nm (log *ε*) : 276 (3.48), 350 (2.77).

*2-[(E)-2-[4-(1H-Benzo[d]triazole-1-yl)phenyl]vinyl]-1,3-benzothiazole-3-oxide* (**1f**)*:* Yield 35%; M.p 219–223 °C; ^1^H-NMR: (DMSO-d_6_) 8.13–8.18 (1H, m, H-7), 8.01–8.05 (1H, m, H-4), 7.92 (1H, d, *J =* 16.3, H-β), 7.89 (2H, d, *J =* 8.5, H-2`,6`), 7.88 (1H, d, *J =* 16.3 H-α), 7.75 (2H, d, *J =* 8.5, H-3`,5`), 7.62–7.70 (6H, m, H-5,6, H_benzotri_); ^13^C-NMR (DMSO-d_6_) δ 145.67, 144.90, 141.71, 135.33, 134.16, 132.77, 131.76, 129.09, 128.60, 127.67, 127.34 (2C), 126.80, 124.62, 123.63, 122.49 (2C), 119.60, 116.60, 115.51, 110.87;UV-vis: λ_max_ /nm (log *ε*): 304 (3.48), 356 (2.82). 

*2-[(E)-2-[4-(Morpholine-1-yl)phenyl]vinyl]-1,3-benzothiazole-3-oxide* (**1g**): Yield 49%; M.p 240–244 °C; ^1^H-NMR (DMSO-d_6_) δ 8.10–8.13 (1H, m, H-7), 7.94–7.97 (1H, m, H-4), 7.72 (1H, d, *J =* 16.5, H-β), 7.54–7.69 (4H, m, H-5,6,2`,6`), 7.52 (1H, d, *J =* 16.2, H-α), 7.00 (2H, d, *J =* 8.7, H-3`,5`), 3.74 (4H, m, OCH_2_), 3.28 (4H, m, NCH_2_); ^13^C-NMR (DMSO-d_6_) δ 151.79, 143.37, 142.95, 136.24, 135.97, 128.87, 127.98, 126.64, 125.78, 123.60, 116.47, 114.43 (2C), 110.64, 65.69, 46.37; UV-vis: λ_max_ /nm (log *ε*): 316 (2.77), 420 (3.41). 

*2-[(E)-2-[4-(Imidazole-1-yl)phenyl]vinyl]-1,3-benzothiazole-3-oxide* (**1h**): Yield 50%; M.p. 196–198 °C**;** ^1^H-NMR (DMSO-d_6_) δ 8.38 (1H, s br, H-2_im_), 8.16–8.19 (1H, m, H-7), 8.00–8.04 (1H, m, H-4), 7.93 (1H, d, *J =* 16.5, H-β), 7.92 (2H, d, *J =* 8.7, H-3`,5`), 7.85 (1H, t, H-4_im_) 7.83 (1H, d, *J =* 16.5 H-α), 7.65–7.68 (2H, m, H-5,6), 7.63 (2H, d, *J =* 8.7 H-2`,6`), 7.13 (1H, t, H-5_im_); ^13^C-NMR: (DMSO-d_6_) δ 142.73, 142.03, 137.08, 135.17, 134.24, 133.89, 131.35, 129.76, 128.64 (2C), 126.97, 125.62, 123.40, 120.27, 117.43, 116.50, 114.67; UV-vis: λ_max_ /nm (log *ε*) : 344 (3.54).

*2-[(E)-2-[4-(4-Methylpiperazine-1-yl)phenyl]vinyl]-1,3-benzothiazole-3-oxide* (**1i**): Yield 63%; M.p. 222–225 °C;^1^H-NMR (DMSO-d_6_) δ 8.10–8.13 (1H, m, H-7), 7.96 (1H, m, H-4), 7.71 (1H, d, *J =* 16.4, H-β), 7.59–7.66 (2H, m, H-5,6), 7.60 (2H, d, *J =* 8.8 H-2`,6`), 7.50 (1H, d, *J =* 16.4 H-α), 6.99 (2H, d, *J =* 8.8 H-3`,5`), 3.25–3.29 (4H, m, NCH_2_), 2.42–2.46 (4H, m, NCH_2_), 2.22 (3H, s, NCH_3_); ^13^C-NMR (DMSO-d_6_) δ 151.41, 142.99, 142.78, 136.53, 135.98, 128.56, 127.61, 126.79, 125.49, 125.10, 116.20, 114.27, 110.18, 54.03, 46.79, 45.29; UV-vis: λ_max_ /nm (log *ε*) : 316 (3.00), 420 (3.57). 

*2-[(E)-2-[4-(4-Etoxycarbonylpiperazine-1-yl)phenyl]vinyl]-1,3-benzothiazole-3-oxide* (**1j**): Yield 50%; M.p. 216–219 °C; ^1^H-NMR: (DMSO-d_6_) δ 8.11–8.14 (1H, m, H-7), 7.95–7.98 (1H, m, H-4), 7.72 (1H, d, *J =* 16.4, H-β), 7.62–7,66 (2H, m, H-5,6), 7.63 (2H, d, *J =* 8.8 H-2`,6`), 7.52 (1H, d, *J =* 16.4 H-α), 7.00 (2H, d, *J =* 8.8 H-3`,5`), 4.07 (2H, q, *J =* 7.0, COOCH_2_), 3.48–3.53 (4H, m, NCH_2_), 3.25–3.30 (4H, m, NCH_2_), 1.20 (3H, t, *J =* 7.0, CH_3_); ^13^C-NMR: (DMSO-d_6_) δ 154.64, 151.46, 143.72, 142.92, 136.35, 133.76, 129.05 (2C), 128.30, 128.11, 125.69, 125.01, 116.53, 114.99 (2C), 110.60, 61.02, 45.74, 42.62, 14.61; UV-vis: λ_max_ /nm (log *ε*) : 276 (3.82), 400 (3,04). 

*2-[(E)-2-[4-(Thiomorpholine-1-yl)phenyl]vinyl]-1,3-benzothiazole-3-oxide* (**1k**): Yield 64%; M.p. 229–231 °C; ^1^H-NMR: (DMSO-d_6_) δ 8.10–8.13 (1H, m, H-7), 7.94–7.97 (1H, m, H-4), 7.70 (1H, d, *J =* 16.5, H-β), 7.60–7.64 (2H, m, H-5,6), 7.60 (2H, d, *J =* 9.0, H-2`,6`), 7.49 (1H, d, *J =* 16.5, H-α), 6.97 (2H, d, *J =* 9.0, H-3`,5`), 3.70–3.73 (4H, m, NCH_2_), 2.61–2.66 (4H, m, SCH_2_); ^13^C-NMR: (DMSO-d_6_) δ 150.43, 143.70, 142.28, 136.34, 131.62, 129.17, 127.93, 127.16. 125.27, 124.71, 116.38, 114.81, 110.10, 58.79, 46.95; UV-vis: λ_max_ /nm (log *ε*): 424 (3.46). 

### Biological testing

The compounds **1c-1h** have been tested for their antimicrobial activities against Gram positive (*Staphylococcus aureus* and *Bacillus subtilis*) and Gram-negative bacteria (*Escherichia coli* and *Pseudomonas aeruginosa*) as well as a yeast *(Candida albicans)* and a mould (*Microsporum gypseum*). The standard plate diffusion method using Mueller-Hinton and Saboraud agar, or standard dilution method in Saboraud medium [14] were used. The tested compounds were dissolved in DMSO. Minimum inhibitory concentrations (MIC in μg/mL) are given in Table 1.

## References

[1] Milne G.W.A. (2005). Gardner´s Commercially Important Chemicals.

[2] Mishra A., Behera R.K., Behera P.K., Mishra B.K., Behera G.B. (2000). Cyanines during 1990s: A review. Chem. Rev..

[3] Michaelidou A.S., Hadjipavlou-Litina D. (2005). Nonsteroidal anti-inflammatory drugs (NSAIDs): A comparative QSAR study. Chem. Rev..

[4] Aiello S., Wells G., Stone E.L., Kadri H., Bazzi R., Bell D.R., Stevens M.F.G., Matthews C.S., Bradshaw T.D., Westwell A.D. (2008). Synthesis and biological properties of benzothiazole, benzoxazole, and chromen-4-one analogues of the potent antitumor agent 2-(3,4-dimethoxyphenyl)-5-fluorobenzothiazole (PMX 610, NSC 721648). J. Med. Chem..

[5] Cho Y., Ioerger T.R., Sacchettini J.C. (2008). Discovery of novel nitrobenzothiazole inhibitors for *Mycobacterium tuberculosis* ATP phosphoribosyl transferase (HisG) through virtual screening. J. Med. Chem..

[6] Zahradník P., Foltínová P., Halgaš J. (1996). QSAR Study of the toxicity of benzothiazolium salts against *Euglena gracilis*: The free-Wilson approach. SAR QSAR Environ. Res..

[7] Magdolen P., Zahradník P. (2000). Foltínová, synthesis, antimicrobial testing and QSAR study of new 2-phenylethenylbenzothiazolium salts substituted by cyclic amines. Pharmazie.

[8] Buffa R., Zahradník P., Foltínová P. (2002). Synthesis and QSAR studies of new conjugated benzothiazole derivatives. Coll. Czech. Chem. Commun..

[9] Zajac M., Hrobárik P., Magdolen P., Foltínová P., Zahradník P. (2008). Donor-π-acceptor benzothiazole derivatives with extended heteroaryl-containing conjugated system: Synthesis, DFT study and antimicrobial activity. Tetrahedron.

[10] Sigmundová I., Zahradník P., Loos D. (2007). Synthesis and study of novel benzothiazole derivatives with potential nonlinear optical properties. Collect. Czechoslov. Chem. Commun..

[11] Hrobárik P., Sigmundová I., Zahradnik P. (2005). Preparation of novel *push-pull* benzothiazole derivatives with reverse polarity: The compounds with potential non-linear optic application. Synthesis.

[12] Takahashi S., Kano K. (1969). Studies on heteroaromatic *N*-oxides. IX. The Synthesis and structure of benzothiazole *N*-oxides. Chem. Pharm. Bull..

[13] Magdolen P., Mečiarová M., Toma S. (2001). Ultrasound effect on the synthesis of 4-alkyl-(aryl)aminobenzaldehydes. Tetrahedron.

[14] Foltínová P., Sutoris V., Blockinger G., Ebringer L. (1978). Antimicrobial effects of some benzothiazole derivatives. Folia Microbiol. (Prague).

